# Comment on "Durable endoscopic gastrojejunal anastomosis using the double-lumen apposing metal stent septotomy technique: Feasibility study"

**DOI:** 10.1055/a-2867-2747

**Published:** 2026-05-12

**Authors:** Giacomo Emanuele Maria Rizzo, Mario Traina, Domenico Lo Gerfo, Pietro Mezzatesta

**Affiliations:** 1 Gastroenterology and Digestive EndoscopyLa Maddalena Cancer CenterPalermoPalermoItaly; 2 18326 ISMETT-IRCCSPalermoSicilyItaly; 3 General and Oncologic SurgeryLa Maddalena Cancer CenterPalermoItaly



Dear Editor,

We read with great interest the article by Kadkhodayan et al. 1 describing the “double lumen apposing metal stent (LAMS) septotomy” technique to achieve a durable endoscopic gastrojejunal (GJ) anastomosis in patients with gastric outlet obstruction(GOO). In this series of six patients, 50% had benign etiology. This concept is particularly appealing for benign GOO (bGOO), where a stent‑independent, durable bypass is crucial and long‑term indwelling LAMS is undesirable 2 . A recent systematic review and meta‑analysis in bGOO showed that standard single‑LAMS EUS‑GJ provides high pooled technical and clinical success (>95% and >93%), with adverse events (AEs) and recurrence rates of 11.6% 3 .

Kadkhodayan et al. reported 100% technical and clinical success, improved nutritional status (mean albumin increase 0.75 g/dL), and no recurrence of obstructive symptoms. Among the four patients who completed both steps, sustained anastomotic patency after stent removal was achieved via spontaneous septal necrosis (n = 2) or planned septotomy (n = 2), without major AEs. These data focus on a durable, stent‑independent endoscopic gastrojejunostomy as feasible endoscopic ultrasound (EUS)‑guided intervention in bGOO. However, some clarification is needed: Benign and malignant etiologies are equally represented, yet only the benign subgroup can meaningfully inform long‑term durability, whereas malignant cases are limited by prognosis. Moreover, three patients (50%) had a preexisting single LAMS placed 12 to 24 months earlier, whereas the others received dual LAMS de novo. Only one underwent simultaneous dual‑LAMS placement, whereas the remaining dual‑LAMS cases had a staged second LAMS after 1 to 3 months. Thus, baseline anatomy and timing of fistula creation are not standardized, complicating extrapolation to a homogeneous benign‑only setting. In addition, the definition and reporting of technical success appear incoherent, because it is defined as the ability “to complete both steps of the procedure without any gross deviation from anticipated procedure steps,” which would conceptually require stent removal with or without septotomy in all patients. Yet only four of six patients (66.7%) underwent Step 2, whereas one was discharged to hospice, and in another, the LAMS were left in situ because of malignant progression. Despite this, technical success is reported as 100% (6/6), suggesting it may have been calculated only on dual‑LAMS placement (Step 1) rather than on the complete two‑step strategy. Clarifying this point would improve methodological clarity. Moreover, there is also procedural variability in stent fixation, because although all sutured LAMS remained in position and one non‑sutured stent migrated, fixation devices and strategy are not specified, limiting generalizability of the migration and safety profile. These issues should be viewed in the context of the growing evidence base for EUS‑GJ in bGOO, where EUS‑GJ offers outcomes at least comparable to surgery and superior to enteral stenting in terms of reinterventions, with the advantages of a minimally invasive approach. Against this benchmark, the double‑LAMS septotomy technique appears as a promising refinement that now requires prospective, etiology‑stratified, comparative evaluation versus standard EUS‑GE and surgical gastrojejunostomy, with uniform technical and outcome definitions.

Emerging long‑term data on indwelling LAMS in mGOO showed that LAMSs are typically left in place without predefined dwell time and are managed on demand, but they undergo progressive luminal narrowing and local tissue reactions over months 4 . Although this approach seems adequate in malignancy, it emphasizes how long‑term LAMS behavior becomes critical when designing durable EUS‑GJ strategies for benign indications and it further supports the rationale for stent‑independent solutions such as double‑LAMS septotomy.

In conclusion, we commend the authors for introducing a novel and conceptually effective strategy to create a durable endoscopic gastrojejunostomy. Their study provides a valuable proof‑of‑concept and a strong rationale for larger, methodologically rigorous trials to determine whether the added complexity of double‑LAMS septotomy translates into meaningful long‑term benefit for patients with bGOO.

**Publication note**

Letters to the editor do not necessarily represent the opinion of the editor or publisher. The editor and publisher reserve the right to not publish letters to the editor, or to publish them abbreviated or in extracts.
